# SVM2Motif—Reconstructing Overlapping DNA Sequence Motifs by Mimicking an SVM Predictor

**DOI:** 10.1371/journal.pone.0144782

**Published:** 2015-12-21

**Authors:** Marina M. -C. Vidovic, Nico Görnitz, Klaus-Robert Müller, Gunnar Rätsch, Marius Kloft

**Affiliations:** 1 Machine Learning Group, Technical University of Berlin, Berlin, Germany; 2 Department of Brain and Cognitive Engineering, Korea University, Anam-dong, Seongbuk-gu, Seoul 136–713, Korea; 3 Memorial Sloan-Kettering Cancer Center, New York City, New York, United States of America; 4 Department of Computer Science, Humboldt University of Berlin, Berlin, Germany; The University of Queensland, AUSTRALIA

## Abstract

Identifying discriminative motifs underlying the functionality and evolution of organisms is a major challenge in computational biology. Machine learning approaches such as support vector machines (SVMs) achieve state-of-the-art performances in genomic discrimination tasks, but—due to its black-box character—motifs underlying its decision function are largely unknown. As a remedy, positional oligomer importance matrices (POIMs) allow us to visualize the significance of position-specific subsequences. Although being a major step towards the explanation of trained SVM models, they suffer from the fact that their size grows exponentially in the length of the motif, which renders their manual inspection feasible only for comparably small motif sizes, typically *k* ≤ 5. In this work, we extend the work on positional oligomer importance matrices, by presenting a new machine-learning methodology, entitled motifPOIM, to extract the truly relevant motifs—regardless of their length and complexity—underlying the predictions of a trained SVM model. Our framework thereby considers the motifs as free parameters in a probabilistic model, a task which can be phrased as a non-convex optimization problem. The exponential dependence of the POIM size on the oligomer length poses a major numerical challenge, which we address by an efficient optimization framework that allows us to find possibly *overlapping* motifs consisting of up to hundreds of nucleotides. We demonstrate the efficacy of our approach on a synthetic data set as well as a real-world *human* splice site data set.

## Introduction

Major technological advances in sequencing techniques within the past decade have facilitated a deeper understanding of the mechanisms underlying the functionality and evolution of organisms. Considering the pure size of a genome, it comes, however, at the expense of an enormous amount of data that demands for automatic and computationally efficient methods in, e.g., genomic discrimination tasks. One of the most accurate approaches to this end consist in the support vector machine (SVM) [1–3] along with the use of a weighted-degree (WD) kernel [4–8], which, in a nutshell, is a similarity measure between two DNA sequences, breaking them into all possible subsequences up to a length *L* and counting the number of matches. The WD-kernel SVM has been shown to achieve state-of-the-art prediction accuracies in many genomic discrimination tasks such as, e.g., transcription start site detection [9]—achieving the winning entry in the international comparison by [10] of 19 competing machine-learning models—and splice site detection [11]. Efficient implementations such as the one contained in the SHOGUN machine-learning toolbox [12], which employs effective feature hashing techniques [13], have been applied to problems where millions of sequences, each containing thousands of nucleotides, are processed at the same time [14].

**Fig 1:**
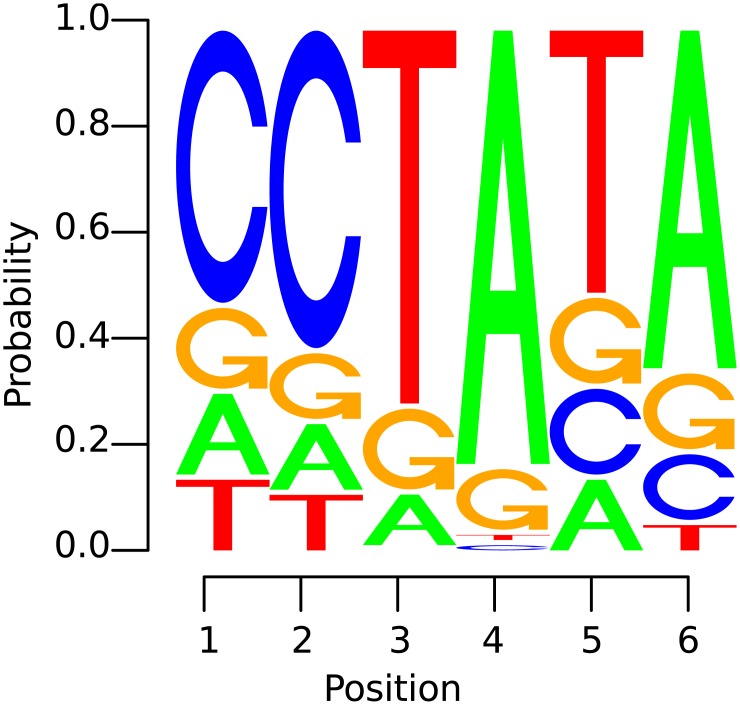
Example of a motif. i.e., an “interesting” subsequence of the DNA—illustrated as a *positional weight matrix* (PWM): the size of a letter indicates the probability of occurrence of the corresponding nucleotide at a certain position in the motif. The likeliest nucleotides are arranged top down.

**Fig 2:**
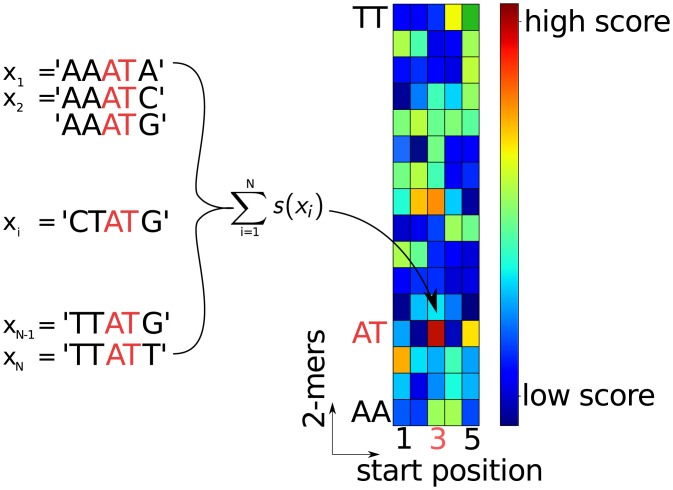
Illustration of a POIM of degree 2 and length *l* = 5 over oligomers of length 2 (“2-mers”). Each POIM entry captures the significance of the particular 2-mer at the specific position in the sequence, which is, roughly said, the expected value of this positional 2-mer regarding the weights in the SVM WD-kernel. Boxes colored in dark red indicate the most discriminative positional 2-mers.

Unfortunately, due to its black-box character, biological factors underlying the SVM’s prediction such as promoter elements and transcription start sites—the so-called *motifs* (illustrated in Fig 1)—are largely unknown. A first step towards the identification of motifs underlying the functionality of organisms is achieved in [15] (for other approaches for interpreting non-linear classification see e.g. [16–20]), where the concept of *positional oligomer importance matrices* (POIMs) is introduced. POIMs assign each *positional oligomer* (PO) *y* of length *l* starting at position *j* with an importance score ${\text{POIM}}_{j,y}∼E\left[s\left(X\right)|X{\left[j\right]}^{l}=y\right]$, which allows us to visualize the significance of the particular POs as illustrated in Fig 2.

Although being a major step towards the explanation of trained SVM models, POIMs suffer from the fact that their size grows exponentially with the length of the motif, which

**Fig 3:**
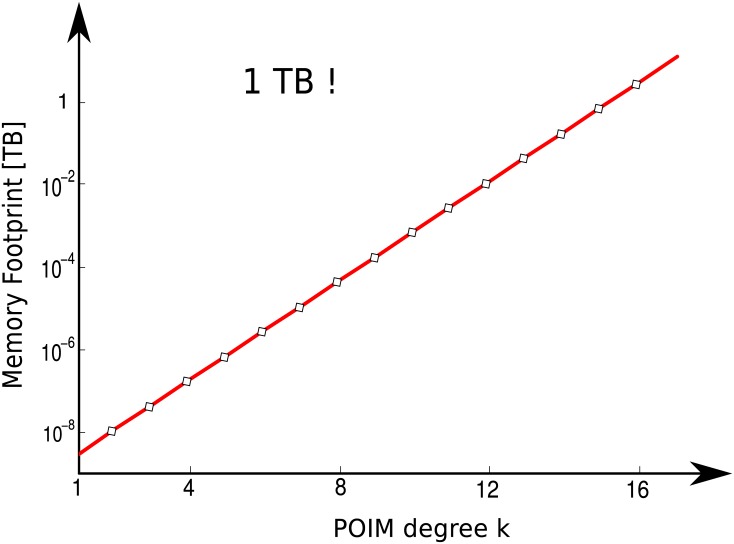
Memory footprint for POIMs of oligomer length *k*. Note that the plot is in semi-logarithmic scale and thus showing an exponential growth for increasing oligomer length rendering a direct approach incomputable for even small *k* ≥ 12.

1. renders their computation feasible only for rather small motif sizes, typically *k* ≤ 12 (see Fig 3 for exemplary execution times)
2. hampers manual inspection (in order to determine candidate motifs) already for rather small motif sizes such as *k* ≈ 5 and is prohibitive for *k* ≥ 10. For example, a POIM of order *k* = 5 contains, at each position, already 4^5^ ≈ 1,000 oligomers that a domain expert has to manually inspect. Slightly increasing the motif length to *k* = 10 leads to an unfeasible amount of 4^10^ ≈ 1,000,000 subsequences per position in the POIM.

**Fig 4:**
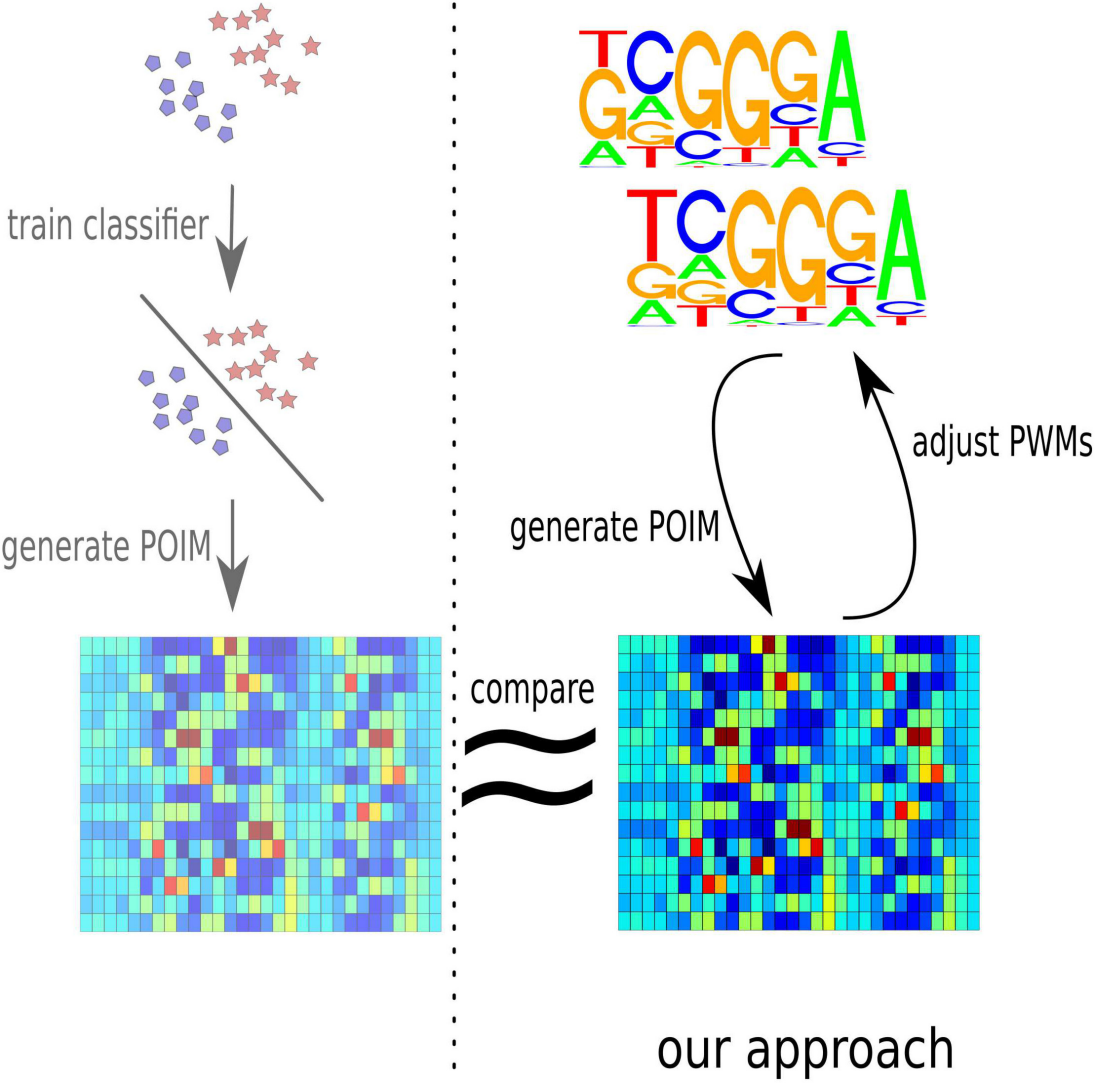
Illustration of the proposed framework to extract motifs from a trained SVM model. In a first step, a POIM is computed corresponding to the trained SVM (shown on the right, from top to bottom). Then a motif approximately corresponding to the POIM is determined by associating each candidate motif (illustrated in the top right) with a motifPOIM (shown in the bottom right) via a probabilistic model and then minimizing the reconstruction error (indicated by a ≈ symbol) by a feedback loop (observe the curved errors on the right) with respect to the truly computed POIM (shown on the bottom left).

In this paper, we tackle the problem of obtaining motifs from the output of an SVM via the use of POIMs from a different perspective. In a nutshell, our approach is the other way round: we propose a probabilistic framework to reconstruct, from a given motif, the POIM that is the most likely to be generated by the motif. By subsequently minimizing the reconstruction error with respect to the truly given POIM, we can in fact optimize over the motif in order to find the one that is the most likely to have generated the POIM at hand. The latter poses a substantial numerical challenge due to the extremely high dimensionality of the feature space. Fig 4 illustrates our approach.

The main contributions of this work can be summarized as follows:

1. Advancing the work of [15] on positional oligomer importance matrices (POIMs), we propose a novel probabilistic framework to finally go the full way from the output of a state-of-the-art WD-kernel SVM via POIMs to the relevant motifs truly underlying the SVM predictions.
2. To deal with the sheer exponentially large size of the feature space associated with the WD kernel, we propose a very efficient numerical framework based on numerous speed-ups such as bit-shift operations, highly efficient scalar multiplications as well as advanced sequence decomposition techniques, and provide a free open-source implementation thereof, which is available at https://github.com/mcvidomi/poim2motif.git.
3. Our approach is able to even find *overlapping* motifs consisting of up to hundreds of nucleotides, while previous approaches are limited to either comparably short or contiguous motifs.
4. We demonstrate the efficiency and efficacy of our approach on both synthetic data sets as well as a *human* splice data set, evaluated by means of the JASPAR database [21].

The paper is structured as follows: after reviewing the traditional approach of obtaining a POIM from a trained SVM model, we introduce the proposed probabilistic methodology—motifPOIM—for approximately determining the motif underlying the observed POIM at hand. Following this, we propose a numerical framework for solving the corresponding non-convex optimization problem by the use of efficient sequence computation techniques such as bit shifts. We evaluate the proposed methodology empirically both on controlled synthetic data as well as real-world *human* splice data. Finally we conclude the paper and discusses starting points for future work.

## Methods

After, firstly defining the weighted degree kernel, we briefly review the positional oligomer importance matrices (POIMs) and then describe our novel approach for extracting motifs from POIMs.

### Preliminaries: Weighted Degree Kernel, Positional Oligomer Important Matrices and Differential POIMs

The weighted-degree kernel, defined as

$$\begin{array}{c} \kappa \left(x,x^{\prime }\right)=\sum_{l=1}^{k}\sum_{j=1}^{L-l+1}I\left\{x{\left[j\right]}^{l}=x^{\prime }{\left[j\right]}^{l}\right\}, \end{array}$$ (1)

breaks two DNA sequences *x* and *x*′ of length *L* into all possible subsequences of length *l* ≤ *L* starting at position *j*, denoted by *x*[*j*]^*l*^ and *x*′[*j*]^*l*^, respectively. The kernel value *κ*(*x*, *x*′) is then obtained by counting the number of matching subsequences, the so-called *positional oligomers* (POs), when traversing the positions *j* = 1, …, *L* − *l* + 1. Equivalently, we may represent the WD kernel by the corresponding binary feature embedding Φ, with *κ*(*x*, *x*′) = 〈Φ(*x*), Φ(*x*′)〉, where each entry of Φ(*x*) represents a valid positional oligomer *y* of length *l* ∈ {1, …, *k*} starting at position *j* ∈ {1, …, *L*−*l* + 1}. A WD-kernel SVM then simply fits the parameter *w* of the linear model *s*(*x*): = 〈*w*, Φ(*x*)〉, which can, more concisely, be expressed as

$$\begin{array}{c} s\left(x\right)=\sum_{l=1}^{k}\sum_{i=1}^{L-l+1}w_{\left(x{\left[i\right]}^{l},i\right)} \end{array}$$ (2)

since Φ(*x*) is inherently *sparse* (only the entries in Φ(*x*) corresponding to the oligomers *y* = *x*[*i*]^*l*^ with *l* ∈ {1, …, *k*} and *i* ∈ {1, …, *L*−*l* + 1} are non-zero).

Let Σ = {*A*, *C*, *G*, *T*} be the DNA alphabet and $X∼U(\Sigma^{L})$ be a uniformly distributed random variable with values in Σ^*L*^ and let *x* ∈ Σ^*L*^ be a realization thereof. For any positional *k*-mer (*y*, *j*) ∈ Σ^*k*^ × {1, …, *L*−*k* + 1} (*k* ∈ {1, …, *L*}), let

$$\begin{array}{c} Q_{k,y,j}:=E\left[s\left(X\right)|X{\left[j\right]}^{k}=y\right]-E\left[s\left(X\right)\right]. \end{array}$$ (3)

The *POIM of order *k** is then defined as the tuple *Q* ≡ *Q*_*k*_: = (*Q*_*k*, *y*, *j*_)_(*y*, *j*)∈Σ^*k*^ × {1, …, *L*−*k* + 1}_. See Fig 2 for an illustration of a POIM of degree *k* = 2. We may interpret Eq (3) as a measure for the contribution of the positional oligomer (*y*, *j*) to the SVM prediction function *s* because a high value of *w*_(*y*, *j*)_, by Eq (2), implies a strong contribution to the prediction score *s*(*x*) if and only if *y* = *x*[*j*]^*k*^. We can very well visualize POIMs in terms of heatmaps as illustrated in Fig 2, from which we may obtain the most discriminative features by manual inspection.

As a first step towards a more automatic analysis of POIMs, [22] propose an extension of the POIM method, the so-called *differential POIM*, which aims to identify the most relevant motif lengths as well as the according starting positions. Formally, the differential POIM Ω is defined as a *k* × *L* matrix Ω: = (Ω_*l*, *j*_) with entries

$$\begin{array}{c} \Omega_{l,j}:=\left\{\begin{array}{c} q_{\max}^{l,j}-\max\left\{q_{\max}^{l-1,j},q_{\max}^{l-1,j+1}\right\}\;\;\text{if}\;l\in \{2,\ldots ,L\}\\ 0\;\;\;\text{elsewise}, \end{array}\right.\; \end{array}$$ (4)

where

$$\begin{array}{c} q_{\max}^{l,j}:=\max_{y\in \Sigma^{l}}\left|Q_{l,y,j}\right|. \end{array}$$

We can interpret Ω_*l*, *j*_ as an overall score for the general importance of the oligomers of length *l* starting at position *j*.

### Extracting Motifs by Mimicking POIMs

In this section, we introduce the proposed motifPOIM methodology for extraction of motifs from POIMs. In a nutshell, it is based on associating each candidate motif by a probability of occurrence at a certain location—which we call *probabilistic positional motif* (PPM)—and then (re-)construct from each PPM the POIM that is the most likely to be generated from the candidate PPM, which we call motifPOIM. The final motif is obtained by optimizing over the candidate motifs such that the reconstruction error of the motifPOIM with respect to the truly given POIM is minimized. See Fig 4 for an illustration.

To this end, let us formally define the PPM as a tuple *m*_*k*_: = (*r*, *μ*, *σ*), where $r\in R^{4\times k}$ and μ,σ∈R. We think of *m*_*k*_ as a candidate motif with PWM *r* and estimated starting position *μ*. The variable *σ* encodes the uncertainty in the location of the motif and can be thought of a standard deviation of the location of the motif. Under this probabilistic model, we define, in analogy to the SVM weight vector *w* occurring in Eq (2), a motif weight vector *v* ≡ *v*(*m*_*k*_) with entries (*v*(*m*_*k*_))_*z*, *i*_ = *v*_(*z*, *i*)_(*m*_*k*_) defined as

$$\begin{array}{c} v_{\left(z,i\right)}\left(m_{k}\right):=\frac{1}{\sqrt{2\pi }\sigma }exp\left(-\frac{{\left(i-\mu \right)}^{2}}{2\sigma^{2}}\right)\prod_{l=1}^{k}r_{z_{l},l}, \end{array}$$

for any positional *k*-mer (*z*, *i*) ∈ Σ^*k*^ × {1, …, *L*−*k* + 1}. Consequently, we define in analogy to Eq (2) a function

$$\begin{array}{c} \bar{s}\left(x|m_{k}\right):=\sum_{i=1}^{L-k+1}v_{\left(x{\left[i\right]}^{k},i\right)}\left(m_{k}\right). \end{array}$$ (5)

By means of the above function, we can construct, from a PPM as defined above, a POIM *R* ≡ *R*(*m*_*k*_) with entries

$$\begin{array}{c} R_{y,j}\left(m_{k}\right):=E\left[\bar{s}\left(X|m_{k}\right)|X{\left[j\right]}^{k}=y\right]-E\left[\bar{s}\left(X|m_{k}\right)\right]. \end{array}$$ (6)

Our overall aim is, by optimizing over the motifPOIM *R*, to approximate the original POIM (cf. also the illustration in the introduction, given by Fig 4). An interesting fact here is that, since computing motifPOIMs for longer PPMs (*m*_*k*_, *k* > 5) is computationally expensive, we may use motifPOIMs of small orders $\tilde{k}\in \left\{2,3\right\}$, although, this is no restriction of the motif length, as we model a PPM of length $k\geq \tilde{k}$ as a number of *D* overlapping SubPPMs, $D:=k-\tilde{k}+1$ with length $\tilde{k}\leq k$. We define the SubPPMs analogous to PPMs as tuples

$$\begin{array}{c} {\tilde{m}}_{d}\left(m_{k},\tilde{k}\right):=\left(\tilde{r},\tilde{\mu },\sigma \right),\;\forall \;d=0,\ldots ,D-1 \end{array}$$

with $\tilde{\mu }:=\mu +d$ and the sub-matrix $\tilde{r}\in R^{4\times \tilde{k}}$ of *r* starting with column *d*.

**Fig 5:**
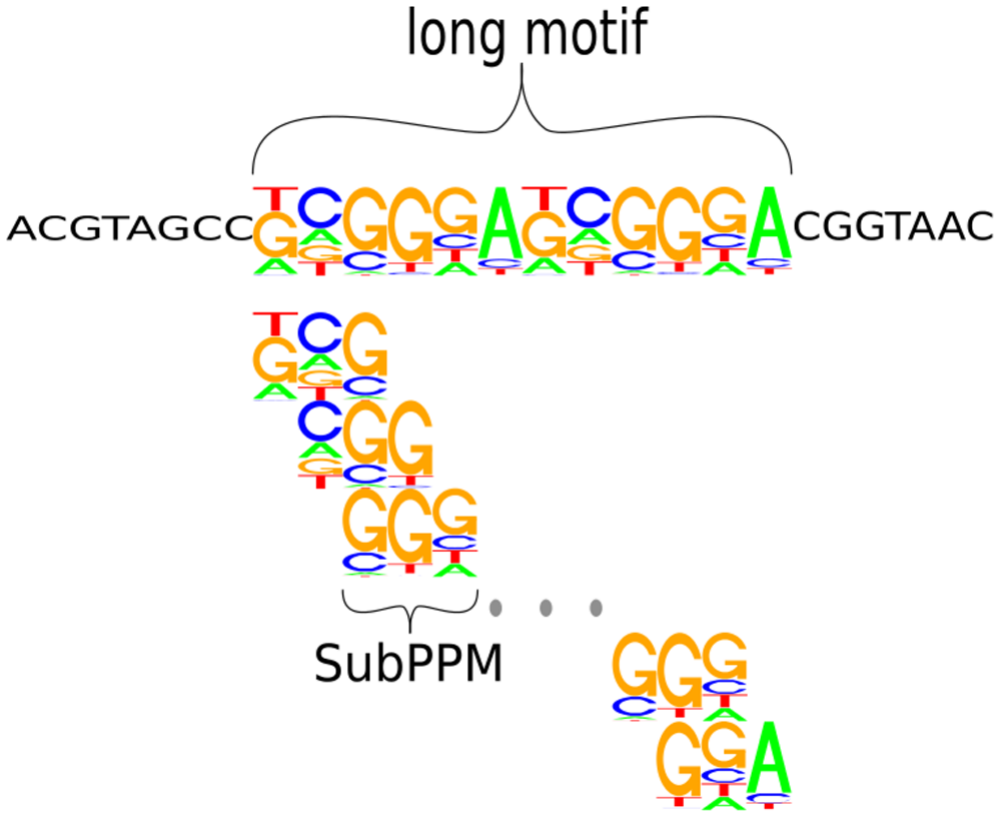
Illustration of the SubPPM approach. instead of computing possibly intractable POIMs for long motifs directly, we decompose each of the longer motifs (here: a single motif of length 12) into smaller overlapping, conforming subsequences of length $\tilde{k}$ (in the figure: $\tilde{k}=3$). This approach allows us to reconstruct motifs of arbitrary length using low dimensional POIMs, rendering the reconstruction of very large, possibly overlapping motifs computationally feasible.

The basic idea is illustrated in Fig 5, where we divide a PPM into a set of SubPPM. Instead of computing an motifPOIM for the PPM, we now compute a set of *D* motifPOIMs for the smaller overlapping SubPPMs.

## Numerical Methods

In this section, we introduce an efficient numerical framework for the extraction of motifs from POIMs by mathematical optimization. The core idea is to determine a motif *m*_*k*_ with an according motifPOIM *R*(*m*_*k*_) that approximates the original POIM *Q*_*k*_. To this end, let us introduce some notation. Let K⊂N be the set of all motif lengths to be considered and $k_{\max}=\max_{k\in K}k$ the maximum length. The vector $T\in N_{0}^{k_{\max}}$ contains the number of PPMs for each motif length, where $T_{k},k\in K$ is the given number of PPMs of length *k*. For example, when K={2,4,10} and *T* = (0, 6, 0, 2, 0, 0, 0, 0, 0, 2), then the goal is to find 6 PPMs of length 2, 4 PPMs of length 4, and 2 PPMs of length 10. Our optimization method is as follows: given the set K and the vector *T*, we randomly initialize the PPMs $m_{k,t}\;t=1,\ldots ,T_{k},k\in K$ and generate a set of motifPOIMs for the SubPPMs ${\tilde{m}}_{d}\left(m_{k},\tilde{k}\right),\;d=0,\ldots ,D-1$. The optimization variables are all $T_{k},k\in K$ PPMs. For obtaining the priorities of the PPMs we weight the PPMs by $\lambda_{k,t},\;t=1,\ldots ,T_{k},k\in K$ and additionally optimize over the weights. Hence, the optimization variables are:

- PPM $m_{k,t}=\left(r_{k,t},\mu_{k,t},\sigma_{k,t}\right),\;t=1,\ldots ,T_{k},k\in K$,
  where
  $$\begin{array}{l} \mu_{k,t}\in \left\{1,\ldots ,L-k+1\right\},\;\;\;t=1,\ldots ,T_{k},k\in K\\ \sigma_{k,t}\in [\epsilon ,k],\;\;\;t=1,\ldots ,T_{k},k\in K\\ r_{k,t}\in {\left[\epsilon ,k\right]}^{4\times k},\;t=1,\ldots ,T_{k},k\in K \end{array}$$
- weight of *m*_*k*, *t*_
  $$\lambda_{k,t}\in \left[0,W\right],\;\;t=1,\ldots ,T_{k},k\in K,W\in R^{+}.$$

A PPM generates a motifPOIM, which is given by the sum of *D* motifPOIMs generated by its SubPPMs. The sum of the weighted motifPOIMs, *λ*_*k*, *t*_ *R*(*m*_*k*, *t*_), *t* = 1, …, *T*_*k*_, should estimate the POIM $Q_{\tilde{k}}$ for each k∈K. The optimization problem is now that of minimizing the distance between the sum of the motifPOIMs and the original POIM, which leads to a non-convex optimization problem with the following objective function:

$$\begin{array}{c} f\left(\eta \right)=\frac{1}{2}\sum_{k\in K}\sum_{y\in \Sigma^{\tilde{k}}}\sum_{j=1}^{L}{\left(\sum_{t=1}^{T_{k}}\lambda_{k,t}\sum_{d=0}^{D-1}R_{y,j}\left({\tilde{m}}_{d}\left(m_{k,t},\tilde{k}\right)\right)-Q_{\tilde{k},y,j}\right)}^{2}, \end{array}$$ (7)

where $\eta ={\left(m_{k,t},\lambda_{k,t},\tilde{k}\right)}_{t=1,\ldots ,T_{k},k\in K}.$

The associated constrained non-linear optimization problem is thus as follows:

$$\begin{array}{c} \min_{{\left(m_{k,t},\lambda_{k,t}\right)}_{t=1,\ldots ,T_{k},k\in K}}f\left(\eta \right)\;\\ \text{subject}\;\text{to}\;\\ \epsilon \leq \sigma_{k,t}\leq k,\;\;\;t=1,\ldots ,T_{k},k\in K\\ 1\leq \mu_{k,t}\leq L-k+1,\;\;t=1,\ldots ,T_{k},k\in K\\ 0\leq \lambda_{k,t}\leq W,\;\;\;\;\;\;t=1,\ldots ,T_{k},k\in K\\ \epsilon \leq r_{k,t,o,s}\leq 1,\;\;\;t=1,\ldots ,T_{k},k\in K\\ \;\;\;\;\;\;o=1,\ldots ,|\Sigma |,s=1,\ldots ,k,\sum_{o=1}^{\left|\Sigma \right|}r_{k,t,o,s}=1 \end{array}$$ (8)

where $W\in R^{+}$. Note that for the sake of optimization efficiency we relax the integer constraint on motifs start positions in the sense that we optimize over positive real numbers. The objective function *f*(*η*) is defined on the compact set *U*, since all parameters are defined in a closed and bounded, convex space. Consequently, if *U* is not empty, *f*(*η*) is a continuously differentiable function, since its conforming parts, that is, the Gaussian function and the product of the PWM entries, all are continuously differentiable. Thus the global minimum of the optimization problem Eq (8) is guaranteed to exist. Due to the non-convex nature of Eq (8), however, there may exist multiple local minima.

### Efficient Computation

To allow an efficient numerical optimization of Eq (8), we first translate the motifPOIM formula Eq (6) in another, equivalent form, similar as in [11]. To this end, note that the expected value of $\bar{s}\left(X|m_{k}\right)$ for the given weight vector *v*(*m*_*k*_) and a random variable $X\in \Sigma^{L}$ is given by:

$$\begin{array}{ccc} E[\bar{s}\left(X|m_{k}\right)] & = & \frac{1}{|\Sigma^{L}|}\sum_{x\in \Sigma^{L}}\bar{s}\left(x;m_{k}\right). \end{array}$$

It holds that

$$\begin{array}{ccc} E[\bar{s}\left(X|m_{k}\right)] & = & \frac{1}{|\Sigma^{L}|}\sum_{x\in \Sigma^{L}}\sum_{l=1}^{k}\sum_{i=1}^{L-l+1}v_{\left(x{\left[i\right]}^{l},i\right)}\left(m_{k}\right)\\ & = & \sum_{l=1}^{k}\sum_{i=1}^{L-l+1}\frac{1}{|\Sigma^{L}|}\sum_{x\in \Sigma^{L}}v_{\left(x{\left[i\right]}^{l},i\right)}\left(m_{k}\right)\\ & = & \sum_{l=1}^{k}\sum_{i=1}^{L-l+1}\frac{1}{|\Sigma^{l}|}\sum_{z\in \Sigma^{l}}v_{\left(z,i\right)}\left(m_{k}\right)\\ & = & \sum_{l=1}^{k}\sum_{z\in \Sigma^{l}}\sum_{i=1}^{L-l+1}v_{\left(z,i\right)}\left(m_{k}\right)P\left(X{\left[i\right]}^{l}=z\right). \end{array}$$ (9)

Hence the conditioned expectation is almost equivalent to Eq (9), except the probability term that is given by the conditioned probability conditioned that *y* is at position *j*:

$$\begin{array}{c} P(X{\left[i\right]}^{l}=z|X{\left[j\right]}^{k}=y). \end{array}$$ (10)

We now consider this probability term and its affect on the summation in Eq (6)). To this end, we introduce the following notation as in [11].

**Definition 1** *Two positional oligomers* (*z*, *i*) *and* (*y*, *j*) *of length* *l* *and* *k* *are independent if and only if they do not share any position; in this case we write* (y,j)⊀(z,i) *and* (*y*, *j*)≺(*z*, *i*) *otherwise (i.e., when they are dependent). If they are dependent and also agree on all shared positions we say they are* *compatible* *and we write* (y,j)≾(z,i) *(and* (y,j)≴(z,i) *if they are not compatible)*.

According to the cases discussed in the above definition, the conditioned probability term can take the following values:

$$\begin{array}{c} \mathbb{P}(X{\left[i\right]}^{l}=z|X{\left[j\right]}^{k}=y)=\;\{\begin{array}{cc} \frac{1}{\left|\Sigma^{l}\right|} & \;\text{if}\;\left(y,j\right)⊀\left(z,i\right)\\ 0 & \;\text{if}\;\left(y,j\right)⋨\left(z,i\right)\\ \frac{\left|\Sigma^{c}\right|}{\left|\Sigma^{l}\right|} & \;\text{if}\;\left(y,j\right)≾\left(z,i\right) \end{array}, \end{array}$$ (11)

where c is the number of shared and compatible positions of two positional oligomers:

$$c\left((y,j),(z,i)\right)=\left\{\begin{array}{cc} l-|i-j| & \;\text{if}\;i<j\;\text{and}\;\left(y,j)≾(z,i\right)\\ l & \;\text{if}\;i=j\;\text{and}\;\left(y,j)≾(z,i\right)\\ k-|i-j| & \;\text{if}\;i>j\;\text{and}\;\left(y,j)≾(z,i\right)\\ 0 & \;\text{else}. \end{array}\right..$$

Taken the case (y,j)⋨(z,i), the probability terms in the motifPOIM formula (6) subtract to zero, so that the positional oligomer (*z*, *i*) is not considered in the sum *R*_*y*, *j*_(*m*_*k*_). Hence, in order to compute *R*_*y*, *j*_(*m*_*k*_), it is sufficient to sum over two positional oligomer sets, where one contains all (*z*, *i*) with (y,j)≾(z,i), $I_{\left(y,j\right)}^{≾}$, and the others contains all (*z*, *i*) with (y,j)⋨(z,i), $I_{\left(y,j\right)}^{⋨}$:

$$\begin{array}{ccc} R_{y,j}\left(m_{k}\right) & = & \sum_{\left(z,i\right)\in I_{\left(y,j\right)}^{≾}}v_{\left(z,i\right)}\left(m_{k}\right)\left(\frac{|\Sigma^{c}|}{|\Sigma^{k}|}-\frac{1}{|\Sigma^{k}|}\right)\\ & & +\sum_{\left(z,i\right)\in I_{\left(y,j\right)}^{⋨}}v_{\left(z,i\right)}\left(m_{k}\right)\left(-\frac{1}{|\Sigma^{k}|}\right)), \end{array}$$ (12)

where $I_{\left(y,j\right)}^{\circ }:=\{\left(z,i\right)\in \Sigma^{\left|y\right|}\times \{1,\ldots ,L-\left|y\right|+1\}|\left(y,j\right)\circ \left(z,i\right)\}$ and ∘∈{≾,⋨}.

#### Numerical Speed-ups

**Fig 6:**
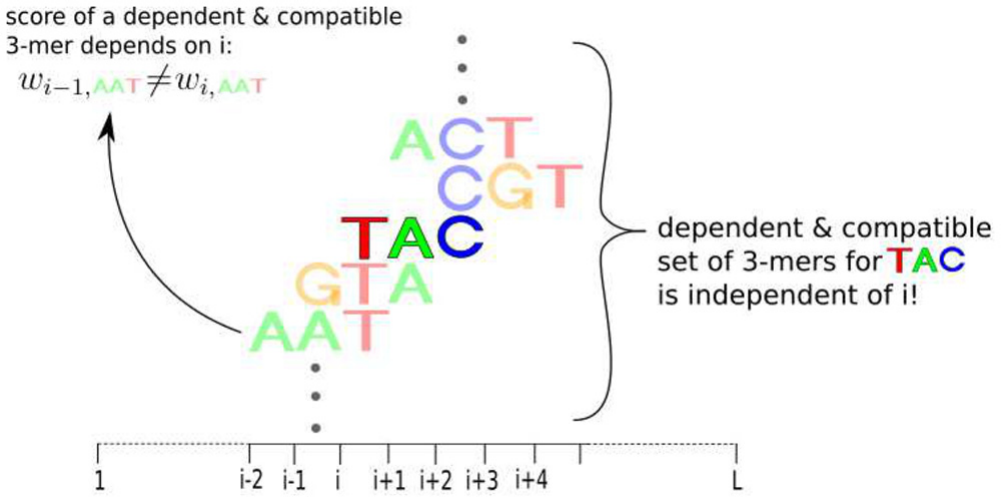
Illustration of the definition of *dependent* and *compatible* oligomers (cf. Definition 1). We say that two positional oligomers are dependent when they overlap each other. If they additionally agree on all shared positions, we say that they are compatible. In this figure, the positional oligomers (*TAC*, *i*) and (*AAT*, *i*−2) are dependent and compatible since both of them contain the letter *T* at position *i*. Whereas the positional 3-mers (*TAC*, *i*) and (*AAG*, *i*−2) are dependent but not compatible.

In addition to the speed-up achieved by the above re-formulation of the problem, we can additionally save time in the motifPOIM computation by exploiting bit shift operations as follows. With the help of the dependence sets $I_{\left(y,j\right)}^{≾}$ and $I_{\left(y,j\right)}^{⋨}$ we know all the dependent and compatible positional oligomers of a single positional oligomer (*y*, *j*). Fig 6 exemplarily illustrates the dependent and compatible oligomers *z* of *y* = *TAC*.

The core idea leading to the numerical speed-up is as follows: In each (*y*, *j*) we consider the two dependence sets. However, the fact is that an oligomer *y* has completely the same dependent and compatible oligomers *z* **at each position** in the sequence. Thus, a dependent set containing all dependent and compatible *z* of *y* is the same for all positions *i* = 1, …, *L*. The trick is to generate a dependency matrix A (see Eq 13) for a single *y* once, which can then be use at every sequence position without the need of recalculation. This matrix contains the probability terms of the motifPOIM formula since they do not change for *y* over the positions, saving at least |Σ^*k*^|(2(*k*−1) + 1) complex computations per position. For each position *j* we now create a weight matrix $C^{j}$ of same size, which contains all the weights *v*_(*z*, *i*)_(*m*_*k*_) for the entries in A for a specific position *j*. Finally, the dot product of A and $C^{j}$ replaces the long motifPOIM formula (12) and we achieve a faster computation speed.

Due to the fact that dependent positional oligomers overlap each other, a dependent *k*-mer *z* of the *k*-mer *y* could have a maximal distance of *k*−1 from *y*. Hence, we have to consider the oligomers *z* with a maximum distance of *k*−1 position next to both sites of *y* and the position of *y* itself. That yields to the dependence set:

$$\begin{array}{c} I_{y}\left(k\right)=\left\{\left(z,i\right)\in \Sigma^{k}\times \left\{1,\ldots ,2\left(k-1\right)+1\right\}\right\}. \end{array}$$

The dependent matrix A(y) is defined on $I_{y}\left(k\right)$ as a matrix of size 4^*k*^ × (2(*k*−1) + 1) and contains the positional oligomer probability terms of the motifPOIM formula as entries:

$$\begin{array}{c} A_{z,i}\left(y\right)=\left\{\begin{array}{cc} \frac{4^{c}-1}{4^{k}} & \;\text{if}\;\left(z,i)≾(y,k\right)\\ \frac{-1}{4^{k}} & \;\text{else} \end{array}\right.. \end{array}$$ (13)

Furthermore, we create a weight matrix $C^{j}$ of same size as *A*, which contains all weights *v*_(*z*, *i*)_(*m*_*k*_) of the entries in A for a specific position *j*, so that the dot product of $C^{j}$ and A replaces the sums of the motifPOIM formula (12), which speeds up computations considerably. This fact is stated in the following theorem.

**Theorem 2** *Let y be a k-mer, m_k_ the PPM, v(m_k_) the motif weight vector, and* A *the dependent matrix of* *y*. *Introducing a matrix* $C^{j}\left(y|m_{k}\right)$, *which is defined on* $I_{y}\left(k\right)$ *as a matrix of same size* Σ^*k*^ × (2(*k*−1) + 1) *as* A(y) *and contains all weights of the positional oligomers in* A(y) *for the motifPOIM position j as*

$$\begin{array}{c} C_{z,i}^{j}\left(y|m_{k}\right)=\left\{\begin{array}{cc} v_{\left(z,i+j-k\right)}\left(m_{k}\right) & \;\text{if}\;1\leq j+i-k\leq L\\ 0 & \;else \end{array}\right., \end{array}$$ (14)

*then*

$$\begin{array}{c} R_{y,j}\left(m_{k,t}\right)=\left\langle A\left(y\right),C^{j}\left(y|m_{k,t}\right)\right\rangle . \end{array}$$ (15)

*Proof*

$$\begin{array}{lll} \langle A(y),C^{j}(y|m_{k,t})\rangle & = & \sum_{z\in \Sigma^{k}}\sum_{i=1}^{2(k-1)+1}C_{z,i}^{j}(y|m_{k,t})A_{z,i}(y)\\ & = & \sum_{z\in \Sigma^{k}}\sum_{i=1}^{2(k-1)+1}v_{\left(z,i+j-k\right)}(m_{k,t})A_{z,i}(y)\\ & = & \sum_{{ℐ}_{\left(y,k\right)}^{≾}}(\frac{4^{c((y,k),(z,i))}-1}{4^{k}})v_{\left(z,i+j-k\right)}(m_{k,t})+\sum_{{ℐ}_{\left(y,k\right)}^{⋨}}(\frac{-1}{4^{k}})v_{\left(z,i+j-k\right)}(m_{k,t})\\ & = & \sum_{{ℐ}_{\left(y,j\right)}^{≾}}(\frac{4^{c((y,j),(z,i))}-1}{4^{k}})v_{\left(z,i\right)}(m_{k,t})+\sum_{{ℐ}_{\left(y,j\right)}^{⋨}}(\frac{-1}{4^{k}})v_{\left(z,i\right)}(m_{k,t}). \end{array}$$

Substituting the last equation into Eq (15) gives us Eq (12).

The case distinction in Theorem 2 is made since some dependent positional oligomers are placed outside the possible sequence positions. Suppose we compute the weight matrix $C_{z,i}^{j}\left(y|m_{k}\right)$ for *y* = *ACT* at the sequence position *j* = 1. Then there are overlapping 3-mers such as, for example, (*AAA*, −1) and (*TAC*, 0), that not exist in the sequence at all. Thus, they are weighted by zero.

Together with the fact that we implement the algorithm in the Python programming language and use the numpy library for computations, calculations are very fast by using the algorithm shown in Table 1.

**Table 1:** Efficient evaluation of Eq (7).

**Data:** $m_{k,t}=\left(r_{k,t},\mu_{k,t},\sigma_{k,t}\right),\lambda_{k,t},\;t=1,\ldots ,T_{k},k\in K$
**Result:** $f{\left(m_{k,t},\lambda_{k,t}\right)}_{t=1,\ldots ,T_{k},k\in K}$
**begin**
*f* ← 0
for k∈K **do**
R←0
**for** *y* ∈ Σ^*k*^ **do**
Compute *A*(*y*) (see Eq 13)
**for** *t* = 1, …, *T*_*k*_ **do**
**for** $j\in I_{CO}$ **do**
Compute *C* ^*j*^(*y*|*m*_*k*, *t*_) see Eq (14)
*R*[*y*][*j*] = *R*[*y*][*j*]+(〈*A*(*y*), *C* ^*j*^(*y*|*m*_*k*, *t*_)〉) (see Eq (15))
**for** *y* ∈ Σ^*k*^
**for** *j* = 1, …, *L* **do**
*f* = *f*+(*R*[*y*][*j*]−*Q*_*k*_[*y*][*j*])^2^ (see Eq 7)

Another step towards an efficient computation is as follows: The probability distribution over the PPM with starting position *μ* in the sequence is a Gaussian function. One characteristic of this function is that 99, 7% of the starting positions are within the confidence interval [*μ*−3*σ*, *μ* + 3*σ*]. Hence, it suffices to compute the motifPOIM entries for the integer values in the confidence interval and set the other motifPOIM entries to zero. Let $I_{CO}$ be the set containing all positional oligomers of the confidence interval. A summary is given in Table 1. For each k∈K a motifPOIM *R* is constructed (see Theorem 2) and the residual between the aforementioned motifPOIM and the SVM POIM *Q*_*k*_ of matching degree *k* is added to the variable iteratively computing the function value.

## Empirical Analysis

In this section, we analyze our proposed mathematical model Eq (8) empirically. After introducing the experimental setup, we evaluate our approach on a synthetic data set where we fully control the underlying ground truth. Finally, we investigate our model on a real *human* splice data set and compare our results to motifs contained in the JASPAR database [21].

### Overall Experimental Setup

For SVM training, we use the shogun machine-learning toolbox [12] (available from http://www.shogun-toolbox.org/), which contains a C++ implementation of a WD-kernel SVM that is specially designed for large-scale sequence learning problems and provides interfaces to matlab, Python, R, and java. The regularization constant *C* of the SVM and the degree *d* of the weighted-degree kernel are set to *C* = 1 and *d* = 20, which are proven default values.

**Fig 7:**
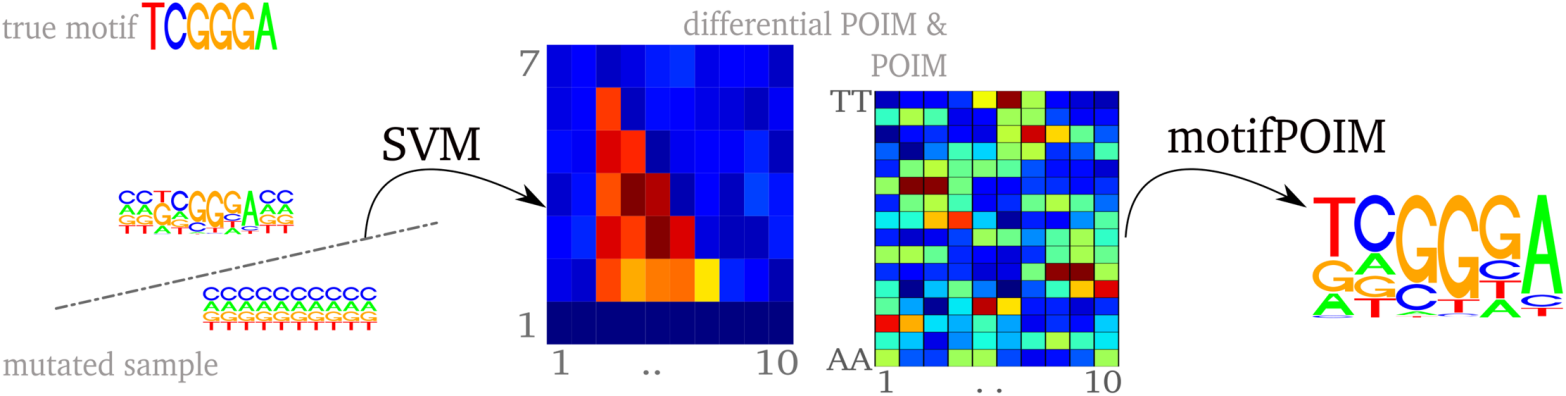
Experimental pipeline of the motif extraction process (from left to right). given a trained SVM, we construct the corresponding POIM before applying the proposed motifPOIM approach to reconstruct underlying motifs (PWMs). Differential POIMs give reasonably initial values for the length and number of motifs.

After SVM training, the POIM *Q* is generated through the Python script compute_poims.py included in the shogun toolbox. The Python framework obtains the trained SVM and a (maximal) POIM degree *k*_*poim*_ = 12 as parameters and returns all POIMs, i.e., the differential POIM, the maximum POIM, and the regular POIMs *Q*_*l*_, *l* = 1, …, *k*_*poim*_. We set *k*_*poim*_ = 7 in synthetic experiments and *k*_*poim*_ = 6 in real experiments because of memory requirements (storing all POIMs up to a degree of 10 requires about 4 gigabytes of space). Note that this is no restriction as our modified optimization problem Eq (8) requires POIMs of degree two or three only. Nevertheless, POIMS of higher degree than three can be provide additional useful information since they contain prior information about the optimization variables, which we use for a proper initialization: For efficient optimization of our highly non-convex optimization problem Eq (8), an appropriate initialization of the optimization variables is mandatory. Thus, we use the differential POIM (defined in Eq (4)) as indicator for extracting the area of interest: we search for points of accumulation of high scoring entries, from which we manually estimate the number of motifs as well as their length and starting position. Thereby we take the whole interval of all highly scoring positions as motif length, where the start position is the first position where all k-mers show a substantial increase in their scores. Once the motif interval is estimated, we select the leading nucleotide from the highest scoring column entry within the interval from the corresponding POIM and initialize the respective PWM entry with a value of 0.7 and 0.1 for non-matches. Indeed, we found this approach to be more stable and reliable than using random initialization. These parameters serve as initialization for our non-convex optimization problem Eq (8). To compute a motif from the computed POIMs, we employ the L-BFGS-B Algorithm [23], where the parameters *λ* and *σ* both are initialized as 1. An illustration of the so-obtained experimental pipeline is shown in Fig 7.

As a measure of the motif reconstruction quality (MRQ), we employ the same score as in JASPAR SPLICE [24]. When comparing equally sized sequences, this scoring reduces to the simple formula

$$\begin{array}{c} \text{MRQ}=\sum_{p=1}^{k}\left[\frac{1}{k}-\frac{1}{2k}\sum_{c\in \{A,C,G,T\}}{\left(t_{cp}-r_{cp}\right)}^{2}\right] \end{array}$$ (16)

### Synthetic Data Experiments

We first evaluate the proposed methodology on synthetically generated data, where we have full access to the underlying ground truth. This experiment aims successive at demonstrating the ability of our method in finding

1. a single motif
2. a single mutated motif
3. overlapping motifs
4. long motifs.

#### Data Sets

To this end, we generate four sample sets *S*_1_, *S*_2_, *S*_3_, *S*_4_ as follows:

1. The sample *S*_1_ consists of 10,000 DNA sequences of length 30 over the alphabet {*A*, *C*, *G*, *T*}^30^, randomly drawn from a uniform distribution $U(\Sigma^{L})$ over Σ^*L*^. We subsequently modify 25% of the sequences by replacing the positions 11 to 16 by the synthetic target sequence CCTATA. These modified sequences form the positively labeled examples, while the remaining 75% of sequences are assigned to a negative label.
2. The sample *S*_2_ is obtained from *S*_1_ by mutating any of the six conforming nucleotides of the inserted motif with probability *p*. This models a scenario where a motif is not quite clearly expressed in the data. We realized the sample $S_{2}\equiv S_{2}^{p}$ for various levels of mutation *p* ∈ [0, 1].
3. Similar to *S*_1_, the sample *S*_3_ consists of 10,000 uniformly drawn DNA sequences of length 30, where, in 12.5% of the sequences, we replace the positions 5 to 15 by the positional oligomer (AATCTGGCGGT, 5). Similarly, we insert the PO (CAATAGCCTGATGGC, 10) into another 12.5% of sequences, resulting in a total of 25% of altered sequences, which are assigned to a positive label (and all other sequences are labeled negatively).
4. The sample *S*_4_ consists of 10,000 uniformly drawn DNA sequences of length 400, where, in 25% of the sequences, we replace the positions 21 to 220 by a positional oligomer of the form *TCGGA TCGGA TCGGA*… with length 200.

### Results

#### Results on the unmutated data set *S*_1_

**Fig 8:**
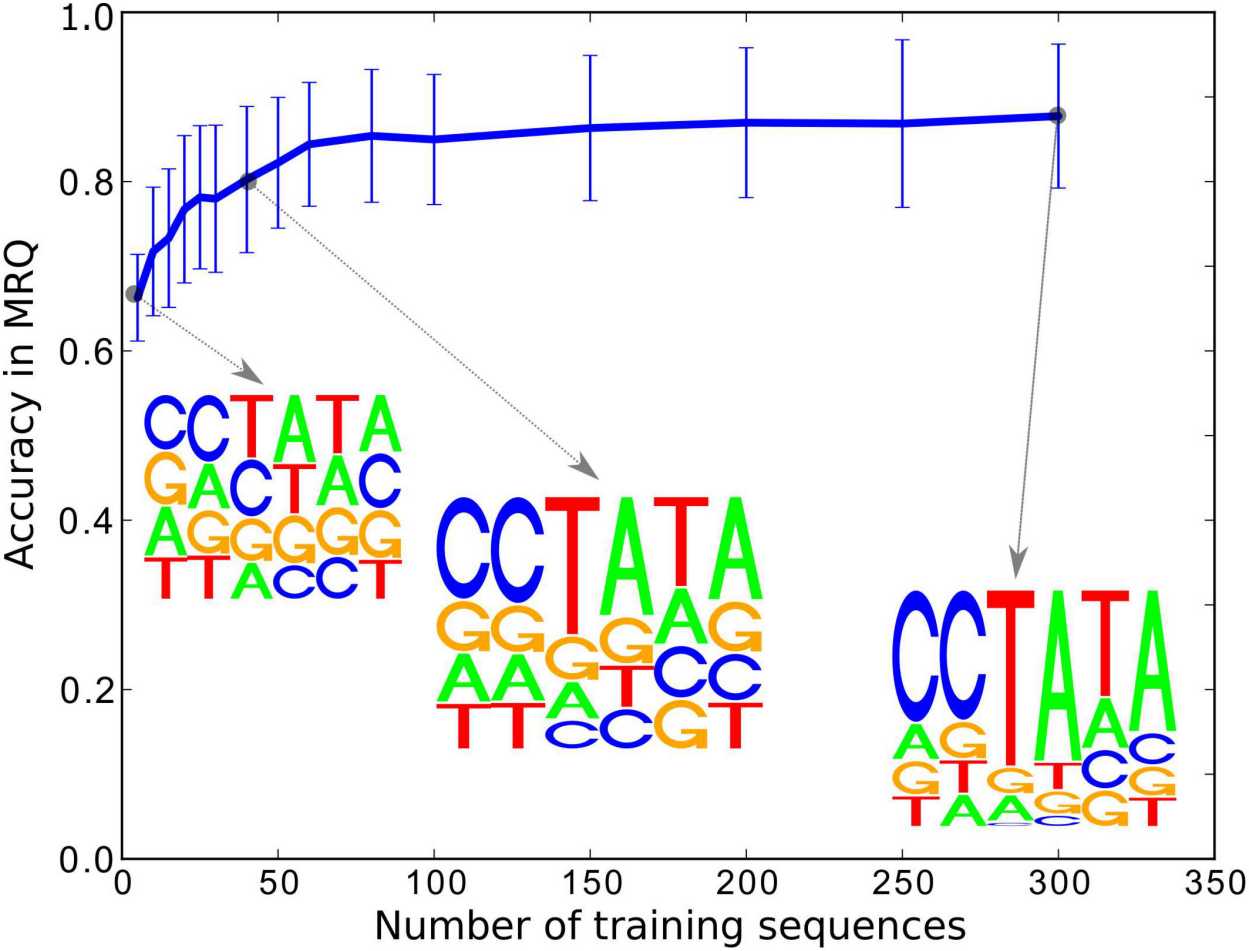
The results of the synthetic experiment for varying SVM training sample size *n* using non-mutated sequences of length 30. As expected, the motif is better reconstructed the more training sequences are used for SVM training. However, as can be seen in the figure, the true motif is picked up early, a tendency that we claim to the robustness of our approach.

The results of the realization of this synthetic experiment using training subsets of size *n* from the base sample *S*_1_ are shown in Fig 8, for various values of *n*. We can observe from the figure that the reconstruction error decreases as a function of the sample size *n* already for *n* = 100. The corresponding motif/PWM computed by our approach correctly identifies the true underlying motif sequence as the most likely path in the PWM.

#### Results on the mutated data set *S*_2_

**Fig 9:**
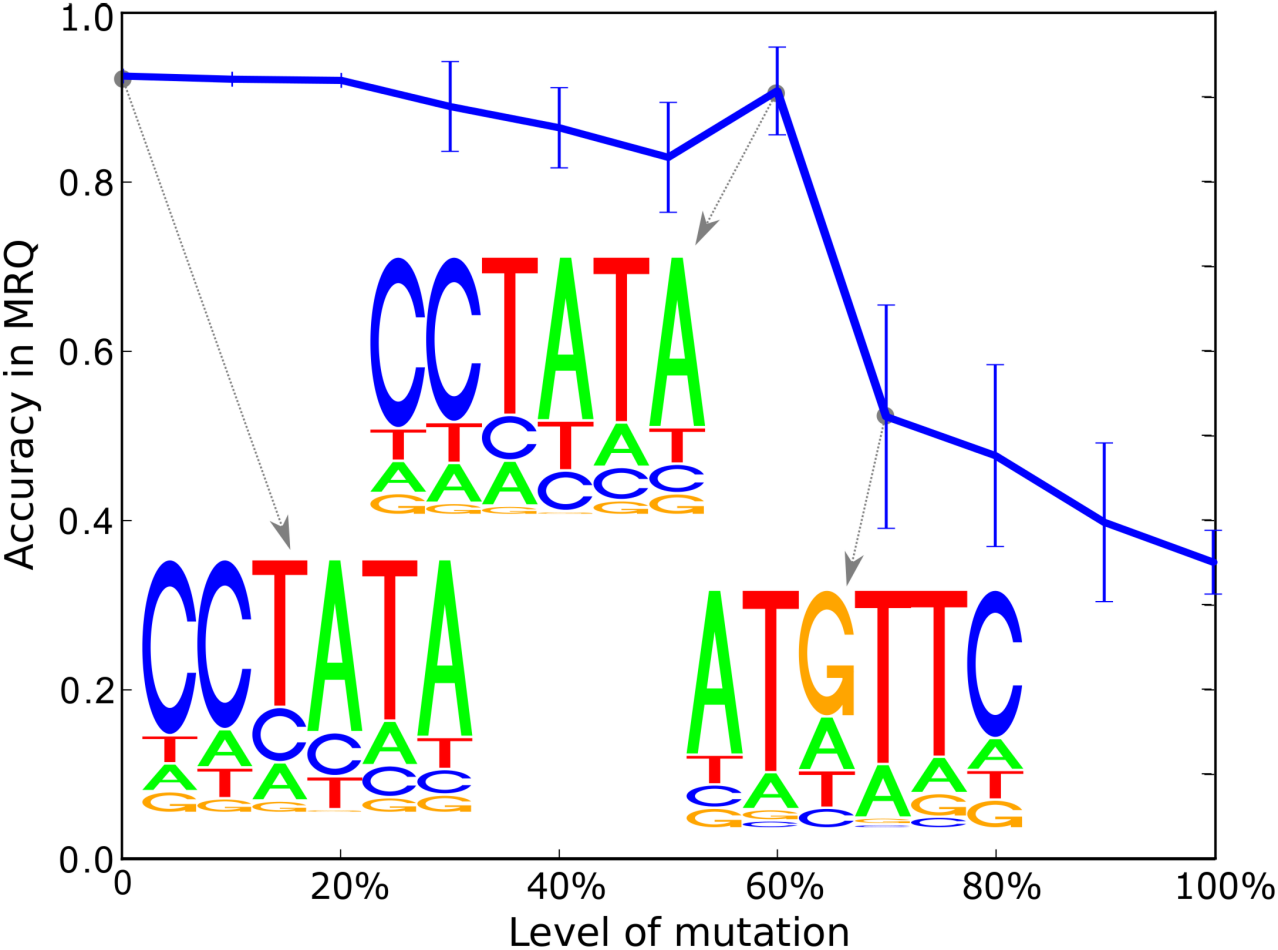
We illustrate the robustness of our approach by plotting the reconstruction errors vs. the mutation level for a fixed amount of training samples. We observe that even for high mutation levels (e.g. 50%) the motif reconstruction quality (MRQ) is sufficiently good to reconstruct the true underlying motif correctly.

Furthermore, we realize the very same experiment using the sample $S_{2}\equiv S_{2}^{p}$ for various levels of mutations. The results are shown in Fig 9. We can observe that, up to a mutation level of 60%, we correctly identify the true underlying motif as being the sequence with the highest probability in the PWM. For more than 70% of mutations in the training data, the performance drops severely. This effect however, is due to a drop of classification performance of the corresponding SVM as can be seen in Table 2. Table 2 highlights results for an exemplary sample for each level of mutation, to relate SVM classification error to mutation level, and also random PWM initialization strategy (30 runs) to greedy initialization.

**Table 2:** Experimental results for a fixed sample *S*_1_ with no mutation (*p* = 0) and *S*_2_ with various levels of mutation (*p* = 0.1, …, 1).

		Greedy PWM init		Random PWM init	
*p*	SVM acc	iter	MRQ	iter	MRQ
0.0	0.9987	14	0.93	39±14	0.8±0.1
0.1	0.998	13	0.92	43±12	0.76±0.12
0.2	0.998	13	0.92	40±19	0.77±0.1
0.3	0.9991	14	0.92	45±21	0.74±0.11
0.4	0.996	13	0.92	41±17	0.8±0.06
0.5	0.9989	14	0.92	36±21	0.79±0.07
0.6	0.9944	13	0.92	41±15	0.78±0.05
0.7	0.616	13	0.46	16±6	0.53±0.08
0.8	0.5	13	0.44	15±2	0.56±0.1
0.9	0.5	14	0.35	15±2	0.55±0.07
1.0	0.5	20	0.33	16±3	0.47±0.08

The proposed greedy initialization of the PWMs is more reliable and stable than randomly initialized PWMs (mean and standard deviations are shown for 30 re-starts), indicated by higher MRQs and less iterations. Furthermore, the SVM classification error is related to the level of mutation and clearly correlated with the motif reconstruction quality (MRQ) of our method, independent of the initialization strategy.

#### Results with overlapping motifs, i.e., data set *S*_3_

**Fig 10:**
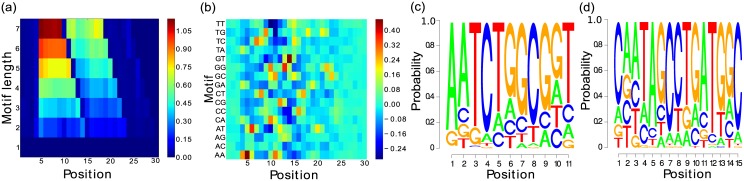
Results for the synthetic experiment with overlapping motifs (AATCTGGCGGT, *μ* = 5) and (CAATAGCCTGATGGC, *μ* = 10). The differential POIM is shown in Figure a), where we can extract the starting position of the two motifs as 5 and 10. Figure b) shows the POIM for the 2-mers, where the area between the starting and ending positions of both motifs is characterized by high scores. Figure c) and d) represent the reconstructed motifs found by our proposed methodology.

To validate our method for overlapping motifs, we also experiment on the sample *S*_3_. The differential POIM and the POIM of degree two resulting from our experimental pipeline are shown in Fig 10(a) and 10(b). Interestingly, the two accumulations of entries with high scores indicate that the POIM includes two overlapping motifs. The investigation of these accumulations is slightly more involved than in the experiment above: we observe, for each motif length *l* > 1, 11−*l* + 1 subsequent cell entries having an extraordinary high score as indicated by light blue, green, orange, or red colors (e.g., length l = 7, we observe a block of 5 subsequent entries). Thus, the first discriminative motif starts at position 5 and consists of 11 nucleotides. We can observe a drop at position 10 (notice the dark blue color) indicating the starting position of the second motif. Altogether, the figure indicates that the optimal model parameters are: K={11,15}, *T*_11_ = 1, *T*_15_ = 1, where *μ*_11, 1_ = 5 and *μ*_15, 1_ = 10. Furthermore, Fig 10 (c) and 10(d) show the PWMs resulting from our optimization approach. We can observe that, although the two motifs are overlapping, both motifs are identified correctly. As for the previous experiment, we also report on the optimal parameters and execution time, shown in Table 3, from which we observe an increase in computation time by a factor of about 5, when contrasted to the runtimes measured on the samples *S*_1_ and *S*_2_. This can be attributed to the presence of multiple motifs in *S*_3_, each having an increased length of 11 and 16 nucleotides, respectively, instead of just 6 nucleotides as in the sample *S*_1_, leading to an increase in computational complexity.

**Table 3:** Execution times and optimal parameters for the synthetic data set *S*_3_ with overlapping motifs.

*μ*	*σ*	*λ*_*opt*_	*f*_*opt*_	time	iter
5	0.77	0.84	0.159	22.68	46
10	0.81	0.68			

Motifs have length 11 and 15 and start at position 5 and 10 respectively. Computational times as well as the number of function evaluations are the same, as our method optimizes holistically everything at once.

#### Results for a very long motif, i.e., data set *S*_4_

**Fig 11:**
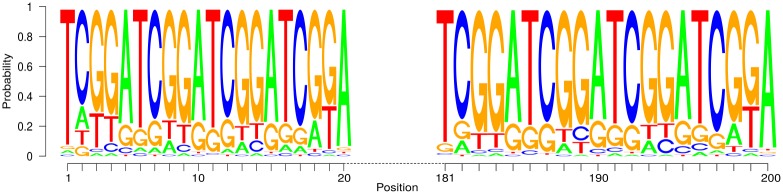
Results of the synthetic experiment on the data set *S*_4_. The motif of length 200 is reconstructed correctly by overcoming the computationally infeasible POIM dimensionality of 4^200^ by splitting the long motif into smaller overlapping motifs. The resulting motif is shown here for the first 20 and the last 181 to 200 positions.

At last, we investigate whether our approach is able to find a very long motif, as contained in the sample *S*_4_. Due to the huge number of variables and the immense size of the POIM, we divide the POIM into 10 smaller conforming parts, in each searching for a motif of length 20. Fig 11 shows the results. We can observe that combination of the 10 computed PWMs reconstructed the real motif adequately.

We can summarize that the experiments on synthetic data demonstrate the ability of our approach to robustly extract the true underlying—possibly overlapping—motifs from noisy data sets even for large motif sizes.

### Application to Human Splice Data

In this section, we evaluate our methodology on a *human* splice data set, which we downloaded from http://www.fml.tuebingen.mpg.de/raetsch/projects/lsmkl. The available human splice dataset contains 15 million samples of length 141, including one percent positive labeled data. For verifying our results we use the JASPAR database [21] (available at http://jaspar.genereg.net), which provides us with a collection of important DNA motifs and also contains a splice site database. As a measure of the motif reconstruction quality (MRQ), we use the JASPAR SPLICE score [24].

Note that real DNA sequences may contain non-polymorphic loci, which is why such a motif is not discriminative and we may thus not expect the SVM to identify this locus. We thus catch this special case and place this positional oligomer in the solution sequence. We apply the full experimental pipeline described in the previous section to the splice data, i.e., we first train an SVM, then generate the POIM and the differential POIM, from which we reconstruct a motif by our motifPOIM optimization approach.

We compare our approach against the publicly available motif finder MEME (Multiple EM for Motif Elicitation, [25]), a well known motif discovering tool for DNA sequences, included in the MEME suite, which is a collection of tools for motif discovering and sequence analyzing. The user can specify the number of motifs as well as the length by either the exact length or a range specification. MEME expects the input sequences in FASTA file format. For comparison, we conducted three experiments with varying numbers of positive samples. For support vector machine training, we double the number of samples by filling in negative ones. We chose 400 positive samples (computation time ∼1min), which is the maximum amount of sequences when using the MEME online tool, 700 positive samples (∼10min), which is the maximum recommended amount when using the MEME locally, and 2000 positive samples (∼12h). We compare the found motifs against the true splice site motif, taken from the JASPAR database with the JASPAR consensus score.

**Fig 12:**
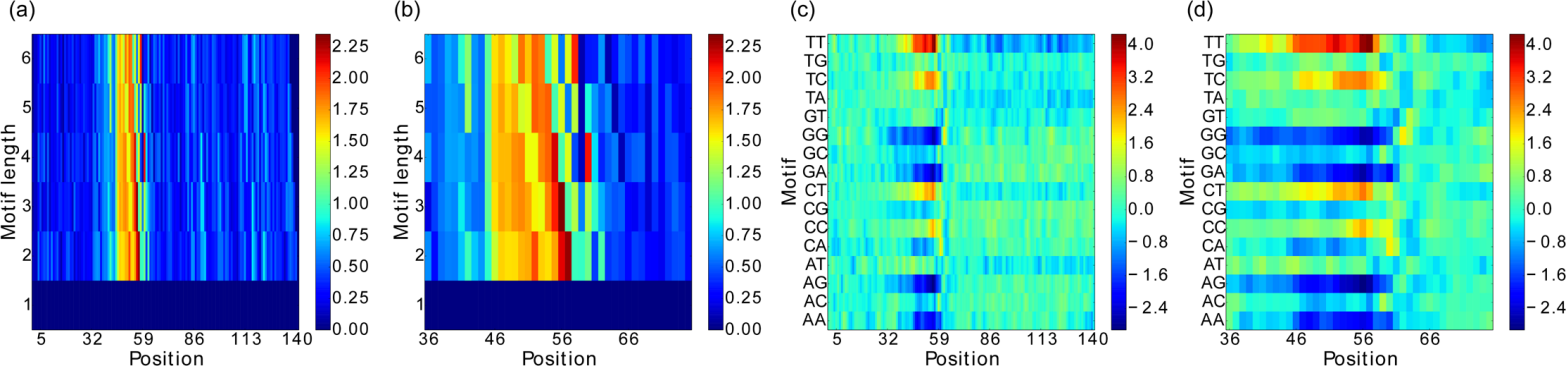
Results of the real-world human splice experiment. Figures (a) and (c) show the differential POIM and the POIM of degree 2, respectively, for the entire sequence length of 200, while Figures (b) and (d) zoom into the “interesting” positions 36–76 only.

Fig 12 shows the preliminary results for 400 samples in terms of the differential POIM and corresponding POIM of degree 2, shown for the entire sequence (see Fig 12 (a) and 12(c), respectively) as well as zoomed in for the “interesting” positions 36–76 of the sequence (see Fig 12 (b) and 12(d)). According to Fig 12 (b), the largest entries correspond to a 3- and 2-mer that can be found at position 56 and 57, respectively. A significant increase of the score is recognizable for all k-mers at position 45, which is enhanced at position 46. The last largest entry for a 6-mer is found at position 58, which corresponds to the last largest entries of 4-mers at position 60 and 2-mers at position 62, from which we conclude that the discriminative motif starts at position 45 and ends at position 63. Thus, the motif we are searching is expected to have a length of 19 nucleotides, which we use as an initialization for our motifPOIM approach. We also account for non-polymorphic loci and find that the nucleotides A and G appear in all DNA sequences of the data set, always at the positions 60 and 61, respectively. We thus place them in the final PWM with a probability of 100 percent.

**Fig 13:**
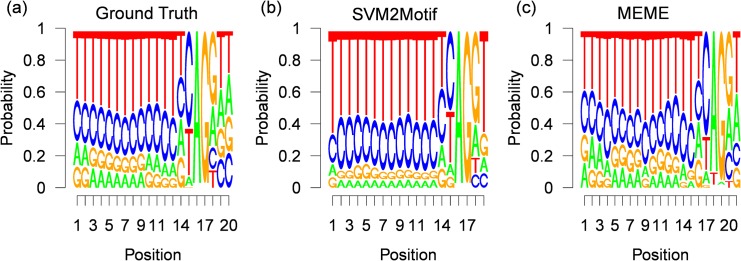
Results for 400 human splice-site examples. Figure (a) shows the (normalized) ground truth motif given by the JASPAR database (20 nucleotides). Figure (b) and (c) depict the corresponding (normalized) PWMs, reconstructed by our approach SVM2Motif (19 nucleotides long, a JASPAR score of 98.92) and by MEME (21 nucleotides, a JASPAR score of 94.77) respectively.

**Fig 14:**
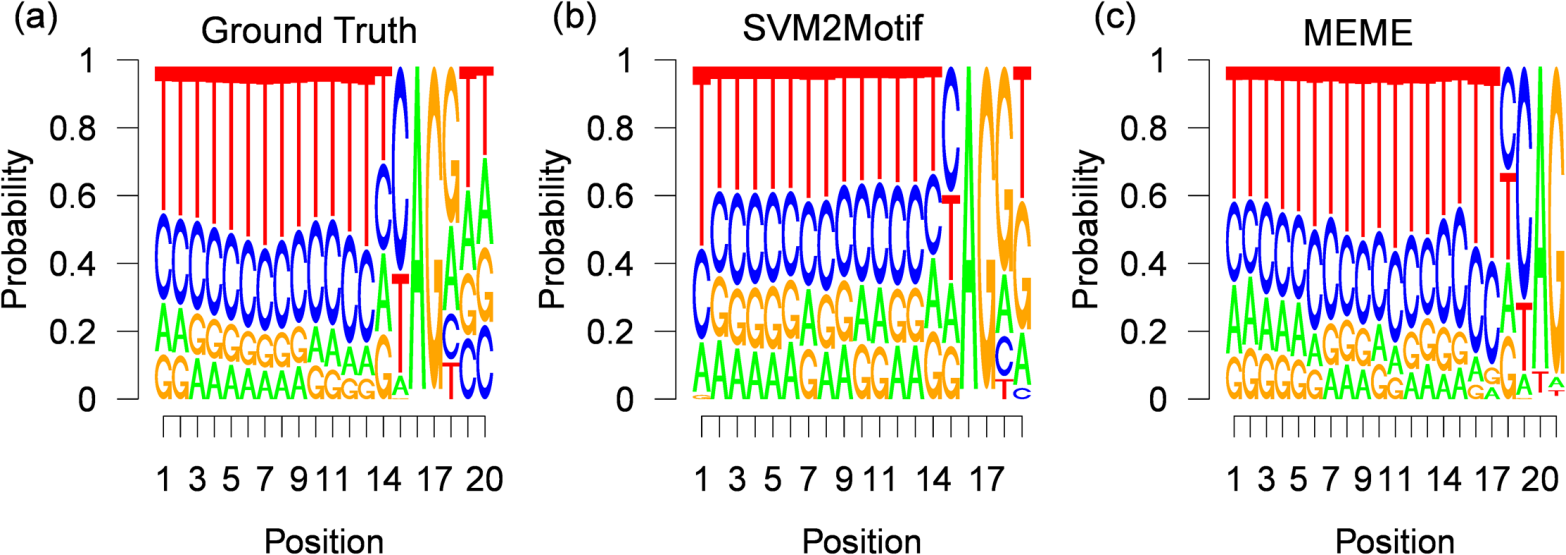
Results for 700 human splice-site examples. Figure (a) shows the (normalized) ground truth motif given by the JASPAR database (20 nucleotides). Figure (b) and (c) depict the corresponding (normalized) PWMs, reconstructed by our approach SVM2Motif (19 nucleotides long, a JASPAR score of 98.51) and by MEME (21 nucleotides, a JASPAR score of 90.06) respectively.

**Fig 15:**
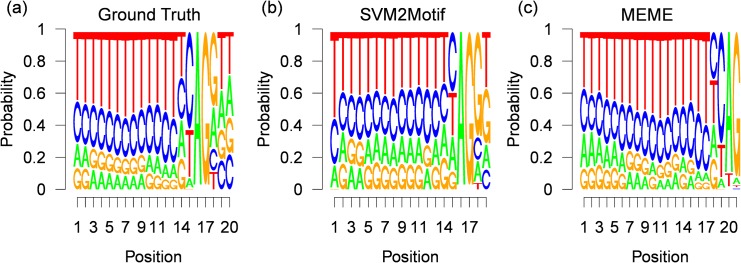
Results for 2000 human splice-site examples. Figure (a) shows the (normalized) ground truth motif given by the JASPAR database (20 nucleotides). Figure (b) and (c) depict the corresponding (normalized) PWMs, reconstructed by our approach SVM2Motif (19 nucleotides long, a JASPAR score of 98.67) and by MEME (21 nucleotides, a JASPAR score of 89.95) respectively.

The final results for 400 positive samples, are shown in Fig 13, where the true underlying motif taken from the JASPAR splice database is shown in Fig 13 (a), while the motif computed by our approach is shown in Fig 13 (b) and the motif found by MEME is shown in Fig 13 (c). The execution times and the optimal parameters found by the L-BFGS-B solver are shown in Table 4. For all experiments, the start position is around the initialization value of 45, with a small variance of up to *σ* = 0.44. The great difference in the optimal function value is caused by the experiment dependent POIM scorings, for example in the POIM of degree 2 of the first experiment we observe a maximal score value of 4 (see Fig 12), where the maximal value in the third experiment was 5. Furthermore, from Table 4, we observe moderate execution times of up to 22.8 seconds. From the resulting motif, shown in Fig 13 (b), we observe a striking accordance with the true motif as evidenced by a high consensus score of 98.6. However, the motif found by MEME, shown in Fig 13 (c), which has a length of 21 nucleotides, has a lower consensus score of 94, 5 although there exists a high similarity to the true motif. The reason is that the motif found by MEME starts 2 positions and ends 1 position before the true motif. The results for 700 and 2000 positive training samples, are shown in Figs 14 and 15, respectively. Here, the results for our approach show similar high consensus scores. MEME, found in both experiments a 21 nucleotides long motif starting 4 positions before the true motif. To get more insights, we fixed the motif length for both methods to 20 nucleotides, which corresponds to the underlying ground truth taken from the JASPAR database. The results are shown in Table 5. Again we observe high consensus scores for the motif computed with our method. Interestingly, the MEME motif finder suffers a severe loss of performance for the first two experiments, achieving consensus scores of around 90 for the last experiment, while the performance of our approach remains comparable. The results show, that our approach is in principle able to infer motifs of high quality and more robust than MEME. Moreover, our approach easily handles sample-sizes beyond MEME.

**Table 4:** Execution times and optimal parameters for the *human* splice data set.

# pos samples	*μ*_*opt*_	*σ*_*opt*_	*f*_*opt*_	time (s)	iter
400	45.0	0.24	175.34	4.19	24
700	44.5	0.44	176.31	22.8	98
2000	44.5	0.4	287.8	16.46	74

**Table 5:** MRQ values for the *human* splice data set.

# pos samples	MEME		SVM2Motif	
	length = 21	length = 20	length = 19	length = 20
400	94.77	90.2	98.92	98.6
700	90.06	88.78	98.51	98.31
2000	89.95	90.4	98.67	97.66

## Conclusion and Discussion

We have developed a new methodology to extract long, overlapping and mutated motifs from trained support vector machines. Putting forward the work of [15] on positional oligomer importance matrices (POIMs), the proposed novel probabilistic framework extracts from the output of a WD-kernel SVM the relevant motifs. To deal with the exponentially large size of the feature space associated with the SVM weight vector and the corresponding POIM (“… we realize that the list of POs can be prohibitively large for manual inspection.” [15], page 8), we proposed a very efficient numerical framework.

The results clearly illustrate the power of our approach in discovering discriminative motifs. In all synthetic data tasks, the hidden motifs could be found and almost perfectly reconstructed. For the human splice site experiments, we recovered known motifs up to a very high precision of 98.39% as compared to the JASPAR Splice data base. A thorough investigation of the association between the found motif and its biological function can be subject to further research.

For practical purposes, a Python framework is available at https://github.com/mcvidomi/poim2motif.git. We have implemented the core algorithms as an add-on to the Python interface of the Shogun Machine Learning Toolbox. It is not only an established machine-learning framework within the bioinformatics community, moreover, it already incorporates the possibility to extract positional-oligomer importance matrices of trained support vector machines with a WD-kernel. Future work will extend our approach to an automatic extraction of the initialization variables, that is, the number of motifs, their length and starting positions. Ultimately, the usage by experimentalists will determine the utility of this approach and govern the direction of further extensions. A core issue might be the extension to other interesting kernels, such as, e.g., spectrum kernels [26], multiple kernels [27–33], other learning methods [34, 35], or learning settings [36–38].
